# Contribution of the Juxtatransmembrane Intracellular Regions to the Time Course and Permeation of ATP-gated P2X7 Receptor Ion Channels*

**DOI:** 10.1074/jbc.M115.642033

**Published:** 2015-04-22

**Authors:** Rebecca C. Allsopp, Richard J. Evans

**Affiliations:** From the Department of Cell Physiology and Pharmacology, University of Leicester, Leicester LE1 9HN, United Kingdom

**Keywords:** ligand-gated ion channel, mutagenesis, ATP, P2X receptor, pore dilation

## Abstract

P2X7 receptors are ATP-gated ion channels that contribute to inflammation and cell death. They have the novel property of showing marked facilitation to repeated applications of agonist, and the intrinsic channel pore dilates to allow the passage of fluorescent dyes. A 60-s application of ATP to hP2X7 receptors expressed in *Xenopus* oocytes gave rise to a current that had a biphasic time course with initial and secondary slowly developing components. A second application of ATP evoked a response with a more rapid time to peak. This facilitation was reversed to initial levels following a 10-min agonist-free interval. A chimeric approach showed that replacement of the pre-TM1 amino-terminal region with the corresponding P2X2 receptor section (P2X7–2Nβ) gave responses that quickly reached a steady state and did not show facilitation. Subsequent point mutations of variant residues identified Asn-16 and Ser-23 as important contributors to the time course/facilitation. The P2X7 receptor is unique in having an intracellular carboxyl-terminal cysteine-rich region (Ccys). Deletion of this region removed the secondary slowly developing current, and, when expressed in HEK293 cells, ethidium bromide uptake was only ∼5% that of WT levels, indicating reduced large pore formation. Dye uptake was also reduced for the P2X7–2Nβ chimera. Surprisingly, combination of the chimera and the Ccys deletion (P2X7–2NβdelCcys) restored the current rise time and ethidium uptake to WT levels. These findings suggest that there is a coevolved interaction between the juxtatransmembrane amino and carboxyl termini in the regulation of P2X7 receptor gating.

## Teaser

**Background:** P2X7 receptor channels show facilitation and pore dilation to repeated applications of ATP.

**Results:** Mutations in either the intracellular amino or carboxyl termini changed the time course and reduced pore dilation. However, when combined, they restored wild-type properties.

**Conclusion:** Interactions between the intracellular termini regulate properties.

**Significance:** This study provides insights into the regulation of channel facilitation and pore dilation.

## Introduction

The widespread role of ATP as an extracellular signaling molecule activating excitatory P2X receptor ion channels has become apparent in the more than 20 years since the initial cloning of the receptors (1). The seven P2X receptor subunits (P2X1–7) have intracellular amino and carboxyl termini, two transmembrane (TM)^2^ segments, and a large extracellular ligand binding loop (2–4). The subunits assemble to form a variety of homo- and heterotrimeric receptors with a range of properties (1, 5). One feature that can be used to distinguish receptor subtypes is the time course of the response. This ranges from rapid desensitization during the continued presence of ATP for P2X1 and P2X3 receptors, P2X2 and P2X4 receptor mediated responses that have a monophasic rise, are relatively sustained (for 10-s agonist application), and readily reproducible, to P2X7 receptors where their initial slow onset kinetics can speed dramatically with repeated stimulation where prolonged or repeated applications are required to reach a steady state response (a process referred to as facilitation or sensitization) (1, 5). The transient activation of P2X1 receptors in platelets contributes to thrombosis (6). P2X2 receptor mutations that render the channel non-functional have been associated with hearing loss (7), and knockout mouse studies have revealed several neuronal roles (for a review, see Ref. 1). P2X7 receptors are expressed on immune cells (8) and have roles in inflammation/pain sensation (9), and responses show plasticity dependent on the duration of stimulation, leading to changes in morphology and, ultimately, cell death (10). Therefore, the temporal characteristics of the receptor are likely to play an important role in their signaling and, therefore, their physiological and pathophysiological roles.

The differences in properties between P2X receptor splice variants has highlighted the contribution of the intracellular amino and carboxyl termini as well as the transmembrane segments to determining the time course of ATP-evoked currents (11–14). This has been supported with work on chimeras; for example, between P2X1 and P2X2 receptors (15). The amino terminus of P2X receptors is similar in length between receptor subtypes and contains a conserved PKC consensus sequence 10–12 residues before a conserved glycine residue at the start of the first transmembrane (TM1) segment. We have shown that, for human P2X1 and P2X2 receptors, this pre-TM1 region between the consensus PKC site and the start of TM1 plays a dominant role in the regulation of the time course and may interact with intracellular carboxyl-terminal residues (16).

The P2X7 receptor is distinct from the other P2X receptor subunits in that it has an intracellular carboxyl-terminal cysteine-rich (Ccys) region between the end of the second transmembrane segment and the conserved Y*XXX*K trafficking motif (17) as well as a C-terminal tail that is >120 amino acids longer (18). The marked facilitation in the time course and amplitude of agonist-evoked responses of P2X7 receptors to repeated applications and dilation of the channel pore (18–20) can be modified by naturally occurring polymorphisms in the C-terminal tail (21), and C-terminal mutations modify pore dilation (18), trafficking (22), and the interaction with other proteins (23). However, the extent to which other variant parts of the receptor contribute to the mechanism underlying the slow initial time course and facilitation is less clear (20). At rodent P2X7 receptors, the P2X7k splice variant, with an alternative N terminus and TM1, shows differences in agonist sensitivity and pore formation (13). This suggests that the amino terminus, and potentially interactions of juxta-TM intracellular amino and carboxyl termini, regulate P2X7 receptor properties.

In this study, we determined the contribution of the pre-TM1 amino-terminal domain and the unique hP2X7 receptor Ccys region to P2X receptor properties by making a series of chimeras (of pre-TM1 amino regions) and insertions/deletions of the Ccys region to human P2X1, 2, and 7 receptors. We have shown that both the amino and Ccys regions are involved in the regulation of the channel time course and large pore formation. Interestingly, mutation of both these regions returns the mutant P2X7 receptor back to wild-type kinetics of activation and pore formation, suggesting that interaction between the intracellular juxtamembrane regions plays an important regulatory role.

## Experimental Procedures

### 

#### 

##### Generation of Chimeric P2X Receptors and Point Mutations

The cDNAs for hP2X1 and hP2X2 receptors have been described previously (16). The hP2X7 receptor was a gift from Dr. Lin-Hua Jiang (University of Leeds, UK). Chimeras were generated by megaprimer-mediated domain swapping, as reported previously (16). In addition to chimeras, point mutants were made using the QuikChange mutagenesis kit (Stratagene). Production of the correct mutations and absence of coding errors was verified by DNA sequencing (Automated ABI Sequencing Service, University of Leicester, UK).

##### Expression in Xenopus laevis Oocytes and Electrophysiological Recordings

Wild-type and mutant constructs were transcribed to produce sense-strand cRNA (mMessage mMachine, Ambion, Austin, TX), as described previously (24). Manually defolliculated stage V *Xenopus laevis* oocytes were injected with 50 nl (50 ng) of cRNA using an Inject+Matic microinjector (J. A. Gaby, InjectMatic, Geneva, Switzerland) and stored at 16 °C in ND96 buffer (96 mm NaCl, 2 mm KCl, 1.8 mm CaCl_2_, 1 mm MgCl_2_, 5 mm sodium pyruvate, 5 mm HEPES (pH 7.6) supplemented with 50 μg/ml gentamycin and 50 μg/ml tetracycline). For electrophysiological recordings (3–7 days post-injection), oocytes were bathed in divalent-free ND96 buffer (96 mm NaCl, 2 KCl, 5 mm sodium pyruvate, 5 mm HEPES, and 0.1 mm flufenamic acid (pH 7.6)). Two-electrode voltage clamp recordings were carried out as described previously (16) in response to U-tube application of ATP (Sigma). The U-tube application system (25) is positioned so that, when activated, the test drug solution flows into/against the flow of the bathing solution. In this way, an interface is formed between the drug solution and the bathing solution. The U-tube was positioned so that the drug/bathing solution interface results in only a small proportion of the oocyte surface being exposed to drug and allows for rapid solution exchange (the drug solution and resulting interface can be visualized by the inclusion of fast green dye to the solution; this has no effect on ATP-evoked responses). By restricting the region of the oocyte exposed to the drug solution, the exchange is more rapid than could be easily achieved for the whole oocyte. It is difficult to directly measure the time course of solution exchange for the U-tube application system. However, with the U-tube system, the (10–50%) rise time of P2X1 receptor currents was 45 ± 9 ms, (*n* = 10). This is not statistically different (*p* = 0.13) from what we recorded with our fast oocyte solution system used for voltage clamp fluorometry recordings (26), which also restricts the area of the oocyte to which test drug solutions are added, where the 10–50% rise time of hP2X1 currents was 29 ± 3 ms (*n* = 39). In the case of the voltage clamp fluorometry perfusion system, we were able to measure the rate of solution exchange around the oocyte with the dye fast green. This had a 10–50% rise time of 8 ± 2 ms (*n* = 8), ∼ 3- to 4-fold faster than the P2X1 receptor current rise time. This suggests that the rate of solution exchange is not the major rate-limiting factor in determining the rise time of the ATP-evoked P2X1 receptor currents. Because we saw no difference in the rise time for ATP currents using the U-tube or the time course-validated voltage clamp fluorometry perfusion system, it seems unlikely that the U-tube application system introduces appreciable errors into the estimate of the time course of the responses. Therefore, the U-tube method is appropriate and has the resolution to allow chimeras/mutations that change the time course of P2X7 receptor currents to be identified.

##### Cell Culture

HEK293-TSA 201 cells were maintained in DMEM supplemented with 10% fetal bovine serum and 1% nonessential amino acids (Invitrogen) at 37 °C in a humidified atmosphere of 5% CO_2_ and 95% air. A monolayer of cells at 80–90% confluence in a 6-well culture dish was transiently transfected using 4 μg of DNA (3.5 μg DNA of targeted receptor and 0.5 μg DNA of GFP) and 10 μl of Lipofectamine 2000 (Invitrogen) in 500 μl of serum-free Opti-MEM1. After 48 h of incubation, cells were plated onto black walled, poly-d-lysine-coated 96-well plates (BD Biosciences) at 100% confluence. Cells were given a minimum of 3 h to adhere to the plate prior to the assay.

##### Dye Uptake

The culture medium was removed, and the cells were washed twice with either reduced divalent normal extracellular solution (149 mm NaCl, 0.8 mm KCl, 0.6 mm CaCl_2_, 0.08 mm MgCl_2_, 10 mm d-glucose, and 10 mm HEPES (pH 7.3)) or sucrose dye uptake buffer (280 mm sucrose, 5.6 mm KCl, 0.5 mm CaCl_2_, 10 mm glucose, 10 mm HEPES, and 5 mm *N*-methyl-d-glucamine (pH 7.4)). Cells were then incubated for 30 min in 100 μl of corresponding buffer containing 20 μm EtBr. EtBr uptake after agonist addition (300 μm, 2′,3′-*O*-(4-benzoylbenzoyl)-ATP, (BzATP, Sigma) was monitored using a fluorometric imaging plate reader (FlexStation) with an excitation wavelength of 525 nm and an emission wavelength of 610 nm (cutoff set at 595 nm). Transfection efficiency was assessed by measuring the cotransfected GFP levels using an excitation wavelength of 395 nm and emission wavelength of 509 nm.

##### Biotinylation of Surface Receptors

Expression levels of wild-type and mutant receptors were estimated by Western blot analysis of total cellular protein and cell surface proteins expressed in both oocytes and transiently transfected HEK293-TSA 201 cells. Injected oocytes/transiently transfected HEK293-TSA 201 cells were treated with 0.5 mg/ml EZ-link sulfo-NHS-LC-biotin (Thermo Fisher Scientific) in ND96/PBS (respectively) for 30 min and washed three times with the corresponding buffer. Oocytes/cells were lysed in 300 μl of buffer H (100 mm NaCl, 20 mm Tris-Cl (pH 7.4), 1% Triton X-100, and 10 μl/ml protease inhibitor mixture (P8340, Sigma)), and 80% of the spin-cleared supernatant (4 °C at 16,000 × *g* for 10 min) was mixed with 30 μl of streptavidin-agarose beads (Sigma) and mixed on a rolling shaker overnight at 4 °C. The remaining 20% supernatant was retained to assess total protein expression for each sample. Beads were washed four times in buffer H, and an equal volume of 2× gel sample loading buffer was added to each and heated to 95 °C for 5 min prior to immunoblotting. Samples were run on a 10% SDS-PAGE gel, transferred to nitrocellulose, and screened for immunoreactivity for the HRP-conjugated anti-FLAG antibody (1:5000, Sigma), anti-P2X1 receptor antibody (APR-001, 1:5000), anti-P2X2 receptor antibody (APR-003, 1:5000), or anti-P2X7 receptor antibody (APR-004, 1:200) (all P2X receptor antibodies from Alomone, Israel). Protein bands were visualized using an ECL Plus kit and Hyperfilm MP (GE Healthcare). For chimeras and Cycs mutants that showed a change in peak current amplitude, there was no significant difference in surface expression from wild-type hP2X receptor levels, with the exception of P2X1–7Nβ, where surface levels were reduced to 16 ± 9% of control P2X1 receptor levels.

##### Statistical Analysis

Data are presented as mean ± S.E., and any differences between the means were determined by one-way analysis of variance followed by Bonferroni's test or Student's *t* test, as appropriate. *n* refers to the number of oocytes or wells of cells tested.

## Results

### 

#### 

##### hP2X7 Receptor Current Facilitation Is Dependent on the Interval between Applications

**FIGURE 1.**
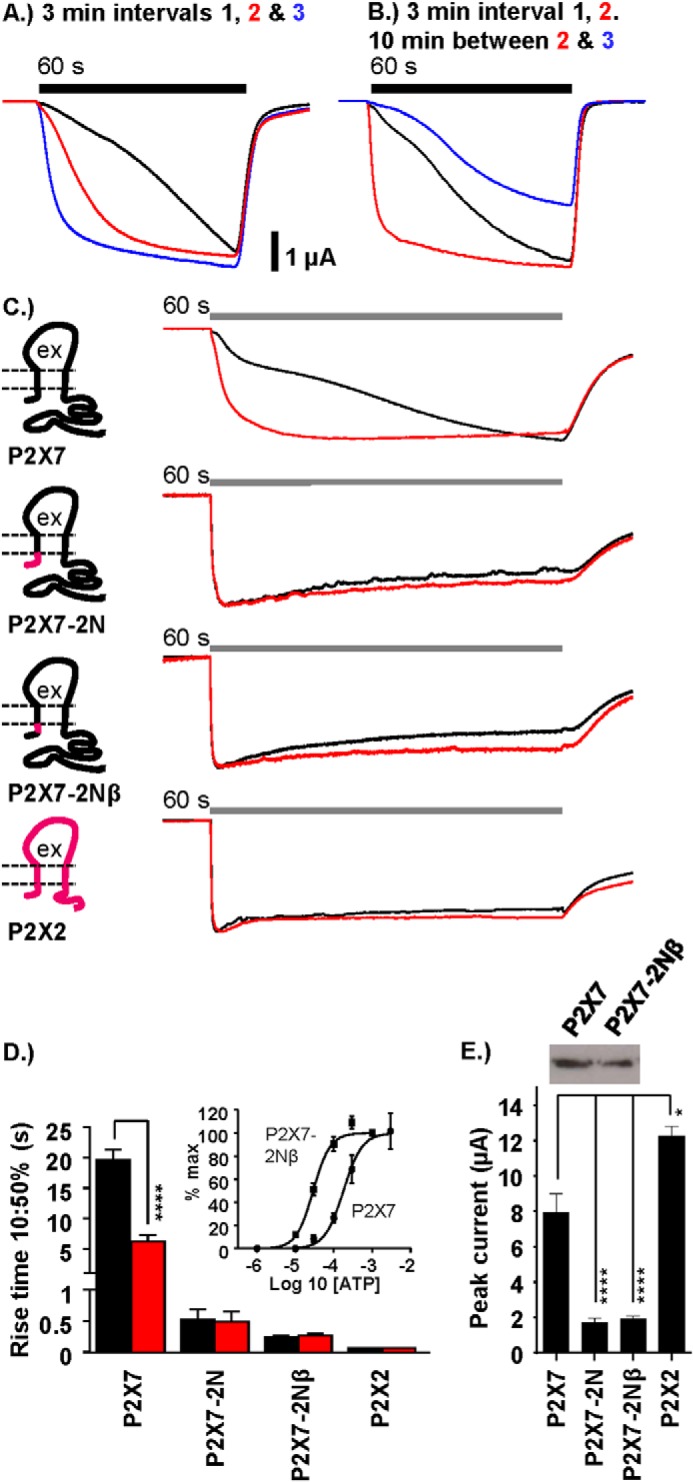
**The pre-TM1 region regulates hP2X7 facilitation.** *A*, representative traces showing the speeding/facilitation of WT P2X7 receptor responses to prolonged (60 s, *bar*) repeat applications of 1 mm ATP at 3-min intervals (*black*, *red*, and *blue trace* consecutively). *B*, increasing the time interval between repeat ATP applications 2 and 3 (*black trace* to *red trace*, 3-min interval; *red trace* to *blue trace*, 10-min interval) returns the receptor response back to its naïve state. *C*, schematics and representative traces showing the effect of replacing the entire N terminus of the hP2X7 receptor (*black*) with that of hP2X2 receptor (*magenta*) or just the 16 pre-TM1 amino acids (*P2X7–2N*β). Traces represent first (*black*) and second (*red*) receptor responses (60-s application at 3-min intervals) to 1 mm ATP. *ex*, the extracellular region of the receptor. *D*, histogram summary showing the 10–50% rise times (seconds) of the first (*black*) *versus* second (*red*) receptor response to EC_90_ ATP (1 mm ATP at P2X7 and 100 μm ATP at P2X7–2N, P2X7–2Nβ, and P2X2) at 3-min intervals. *Inset*, concentration response to ATP for P2X7 and P2X7–2Nβ receptors. *E*, histogram summary showing the peak amplitude (microamperes) of the response to the first ATP application. *Inset*, representative Western blot of the equivalent levels of surface expression of P2X7 and P2X7–2Nβ receptors. Data are mean ± S.E. (*n* = 7–25). *, *p* < 0.05; ****, *p* < 0.0001.

**FIGURE 2.**
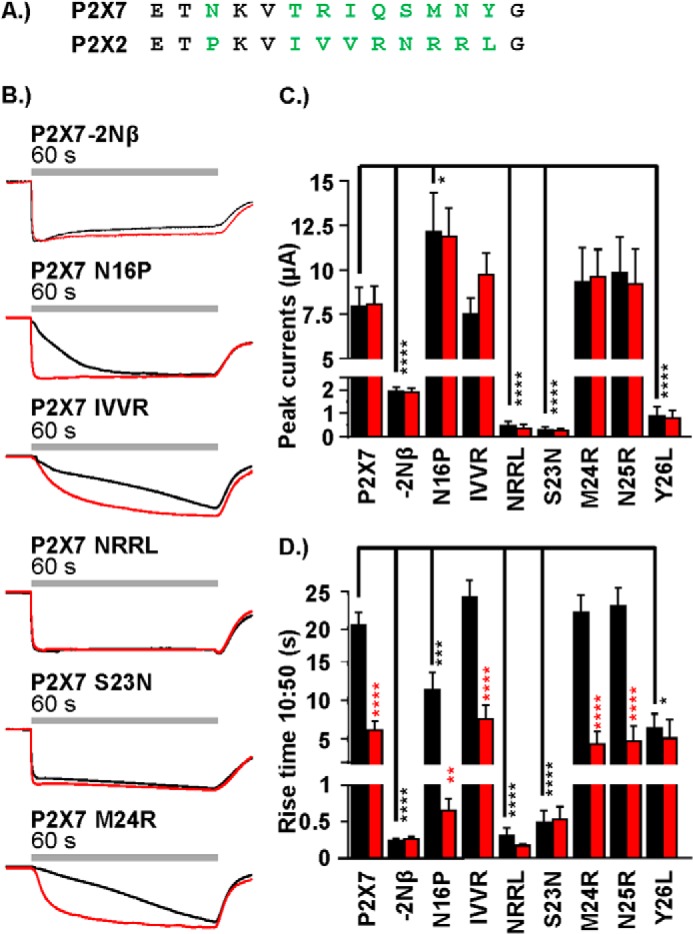
**Contribution of variant pre-TM1 residues to P2X7 receptor current facilitation.** *A*, amino acid sequence lineup showing the pre-TM1 residues of the hP2X7 receptor (*top row*) and the hP2X2 receptor (*bottom row*). Non-conserved amino acids are shown in *green. B*, representative traces (the first and second responses are shown in *black* and *red*, respectively) demonstrating the effect of pre-TM1 substitution of non-conserved amino acid residues between the P2X7 and P2X2 receptor in response to ATP (1 mm, 60-s agonist addition at 3-min intervals). *C* and *D*, histogram summaries showing the peak current amplitude (microamperes and 10–50% rise time (seconds) of the first and second responses to ATP. Statistical significance shown in *black* is relative to the P2X7 WT and, in *red*, for between the first and second response at a particular receptor. Data are mean ± S.E. (*n* = 4–25). **, *p* < 0.01; ***, *p* < 0.001; ****, *p* < 0.0001.

ATP evoked inward currents at the hP2X7 receptor. The initial response to ATP (1 mm EC_90_ concentration) was characterized by a slow rise, and the current generally saturated toward the end of the 60-s agonist application (Fig. 1*A*). The inward current could usually be discriminated into an initial component (10–20 s) and then a brief slowing in rise time/inflection before a secondary rise in amplitude toward a steady-state level. Following ATP washout, the current returned to baseline levels. When there was a 3-min interval between the applications, subsequent responses to ATP had a faster rise time (measured as 10–50% rise time) (Fig. 1*A*) and similar peak amplitude (Fig. 2*C*). These results show that hP2X7 receptor currents show a change in time course, with faster responses to repeated applications, as reported previously (20). This facilitation in time course does not result from an influx of calcium because recordings were made in the absence of extracellular divalent ions. If an interval of 10 min was given between the second and third applications, the time course of the current returned to the initial naïve rate (Fig. 1*B*), showing that the facilitation process is reversible.

##### Contribution of the Intracellular Amino Terminus to hP2X7 Receptor Channel Gating

We have shown previously, using a combination of chimeras and point mutants, that the amino terminus can play an important role in the regulation of the time course of P2X1 and P2X2 receptor currents (16). ATP (100 μm EC_90_ concentration) application at the hP2X2 receptor evoked a rapidly rising current (10–50% rise time 71 ± 6 ms) that showed ∼10% decay during the 60-s agonist application (87 ± 3% of peak current remaining at the end of the 60-s pulse) (Fig. 1*C*), equivalent to that reported previously (16). We were therefore interested to determine the effect of replacing the amino terminus of the facilitating hP2X7 receptor with that from the hP2X2 receptor (Fig. 1*C*). At this chimera (P2X7–2N), ATP-evoked (100 μm) responses had a similar time course as the hP2X2 receptor, with a rapid rise time (10–50% rise time, 459 ± 158 ms) ∼40-fold faster than the first response of WT P2X7 and ∼12-fold faster than the facilitated P2X7 response. The time course, amplitude, and desensitization was the same for a second application of ATP 3 min later. These results indicate that the amino terminus contributes to the control of P2X7 receptor gating and facilitation. We showed previously that, within the amino terminus, it was the residues closest to the first transmembrane segment that made the most important contribution to the regulation of the time course (16). We therefore tested whether swapping the variant region between the conserved consensus sequence for protein kinase C and the conserved glycine before TM1 alone (residues 14–26) could speed the response to ATP. Interestingly, swapping of the Nβ region increased ATP sensitivity ∼8-fold (pEC_50_ of 3.7 ± 0.01 and 4.5 ± 0.01 for WT and P2X7–2Nβ, respectively). To account for this change in sensitivity, the time course of response for the chimera was determined for an EC_90_ concentration of ATP (100 μm, 10-fold lower than for the WT). This rise time at the P2X7–2Nβ chimera was essentially the same as that of the P2X7–2N chimera and similar to the P2X2 receptor. The peak current amplitude at the P2X7–2Nβ chimera was only a quarter that of the P2X7 WT receptor. This did not result from a change in channel expression because the chimera was expressed at similar levels as the WT receptor at the cell surface (Fig. 1*E*) and indicates that the reduction in amplitude results from an effect on channel gating.

The rise of the current at the P2X7 receptor often showed an initial inflection (Fig. 1). This raised the possibility that only the first component was recorded for the P2X7–2Nβ chimera or that a threshold current amplitude needed to be exceeded to trigger the secondary facilitating current. However, this appears not to be the case because, when the traces were normalized to the amplitude at the inflection point, the P2X7–2Nβ currents were still faster, and, when P2X7 currents (recorded after a shorter period of time after RNA injection to reduce channel expression) were of similar peak amplitude as P2X7–2Nβ, they still showed the slow growth of current to the initial application (10–50% rise time of 21.2 ± 2.7 s and 19.8 ± 1.9 s for WT P2X7 receptor currents that were <3 and >6 μA, respectively). Taken together, these results highlight the fact that the residues before TM1 are important for controlling the unique gating features (low ATP sensitivity and time-course/current facilitation) of the P2X7 receptor.

##### Contribution of Variant Residues in the Pre-TM1 Region to P2X7 Receptor Properties

The juxtamembrane amino-terminal Nβ region contains 11 residues, of which nine are variant between the P2X7 and P2X2 receptors (Fig. 2*A*), an N-P variation within the consensus sequence for protein kinase C phosphorylation (T*X*K), and a further eight residues before the conserved glycine at the start of TM1. Mutations were made to determine the contribution of the variant residues. The P2X7-N16P mutant had no effect on peak current amplitude but resulted in an ∼50% speeding up of the time course of the initial response, and subsequent responses were as fast as those of the P2X7–2Nβ receptor (Fig. 2). Changing the four variant residues TRIQ to IVVR (19–22) had no effect on either the peak current amplitude or the time course of the response (compared with P2X7 WT, Fig. 2). In contrast, swapping the four residues SMNY to NRRL (23–26) reduced peak current amplitude by >95%, to 0.49 ± 0.17 μA, and ATP-evoked responses had the fast, reproducible time course of the P2X7–2Nβ chimera (Fig. 2). To determine the contribution of the individual variant residues in this region, we made single point mutants. The P2X7-M24R and N25R mutants were indistinguishable from the P2X7 WT (both amplitude and time course, Fig. 2). The P2X7-S23N mutant had considerably reduced peak current levels (<5% of P2X7, *p* < 0.0001) and the reproducible time course of the P2X7–2Nβ chimera (Fig. 2). The P2X7-Y26L mutant had reduced current amplitudes (∼10% of control, *p* < 0.0001) and a modest, 2.7-fold speeding up of the first response to ATP (*p* < 0.0001). However, the rise time of the second application to ATP had the same time course as the WT P2X7 receptor. These results suggest that the change in properties at the P2X7–2Nβ chimera were dominated by the S23N mutation close to TM1, with a contribution from the variation N16P.

##### Reciprocal Amino-terminal Chimeras

**FIGURE 3.**
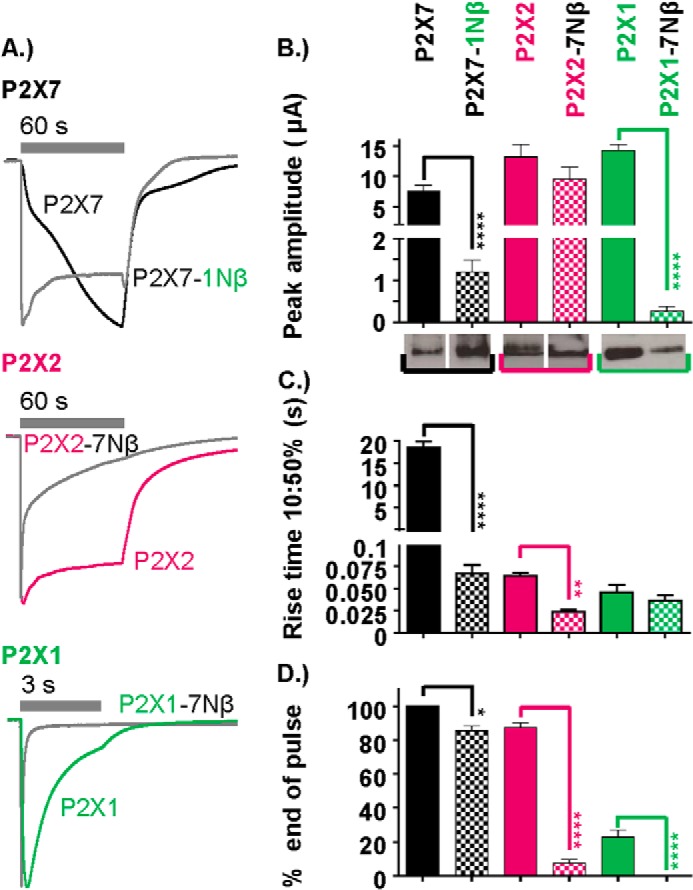
**Contribution of the pre-TM1 region to the time course of P2X1, P2X2, and P2X7 receptor currents.** *A*, normalized traces demonstrating the effect on the time course of pre-TM1 β-region substitution of P2X1 into P2X7, P2X7 into P2X2, and P2X7 into P2X1 receptors, respectively. In each case, ATP was applied for the duration indicated by the *gray bars* (1 mm for P2X7 and P2X7–1Nβ and 100 μm for P2X1 and P2X2 WT receptors and chimeras). *B—D*, histograms showing the peak current amplitude (microamperes), 10–50% rise time (seconds), and percent current remaining at the end of the ATP pulse (EC_90_ concentration of ATP applied for the duration as indicated on the adjacent traces). Western blots show the surface expression of the respective WT and chimeric receptors. For P2X2 and P2X7 receptors, the lanes are from the same blot and exposure, and the *white line* between them indicates that they have been reordered from that loaded on the gel. Data are mean ± S.E. (*n* = 4–25). *, *p* < 0.05; **, *p* < 0.01; ****, *p* < 0.0001.

The chimeras and point mutations identified two variant residues between the P2X2 and P2X7 receptors in the pre-TM1 region that have an effect on time course. The P2X1 receptor region shares the residues corresponding to Pro-16 and Asn-23. We therefore tested whether swapping the Nβ region of the P2X7 receptor with that from the rapidly rising and desensitizing P2X1 receptor had any effect on the time course. The P2X7–1Nβ chimera showed an increase in rise time and was faster than for P2X7–2Nβ (67 ± 9 ms and 118 ± 15 ms, respectively, 10–50%, *p* < 0.01). However, in contrast to the P2X7–2Nβ, there was a modest, ∼15% decay during the 60-s agonist application (Fig. 3). Like P2X7–2Nβ, the P2X7–1Nβ chimera had reduced peak current amplitude (1198 ± 282 nA, Fig. 3) but no change in surface expression. These results support the hypothesis that residues 16 and 23 make an important contribution to the time course of responses.

Because facilitation of the P2X7 receptor could be removed by swapping the pre-TM1 region with a section from either a desensitizing (P2X1) or non-desensitizing (P2X2) receptor, it raised the possibility that the P2X7 pre-TM1 region imparts the facilitatory/slow rise time phenotype. To test whether this was the case, we generated reciprocal chimeras, inserting the P2X7 pre-TM1 region into either P2X1 (P2X1–7Nβ) or P2X2 (P2X2–7Nβ) receptors (Fig. 3). Neither of these reciprocal chimeras showed a slow rise time/facilitation. Indeed, swapping the pre-TM1 region for that from the P2X7 receptor actually increased desensitization at both P2X1 (decay to 50% peak current, 45 ± 7 ms and 1348 ± 136 ms for P2X1–7Nβ and P2X1, respectively) and P2X2 receptors (P2X2–7Nβ decay to 50% of peak current in 1475 ± 327 ms with 7.5 ± 2.2% and 88 ± 2.5% remaining at the end of the 60-s pulse for P2X2–7Nβ and P2X2 WT, respectively). For the P2X1–7Nβ chimera, the peak amplitude of the currents was also reduced by >97%, and the reduction in total charge flow through the open channel was even greater. Because the P2X1 receptor enters the desensitized state through the agonist-bound open state (27), this indicates a marked effect on channel gating. The reduction in current was associated with an 84 ± 9% decrease in surface expression of the receptor, indicating that reduced trafficking contributes to the reduction in current amplitude. The results also show that the juxtamembrane amino terminus is important for the regulation of channel gating, and it is unlikely that the hP2X7 Nβ region alone is responsible for current facilitation.

##### Contribution of the hP2X7 Receptor C-terminal Cysteine-rich Region to Current Facilitation

**FIGURE 4.**
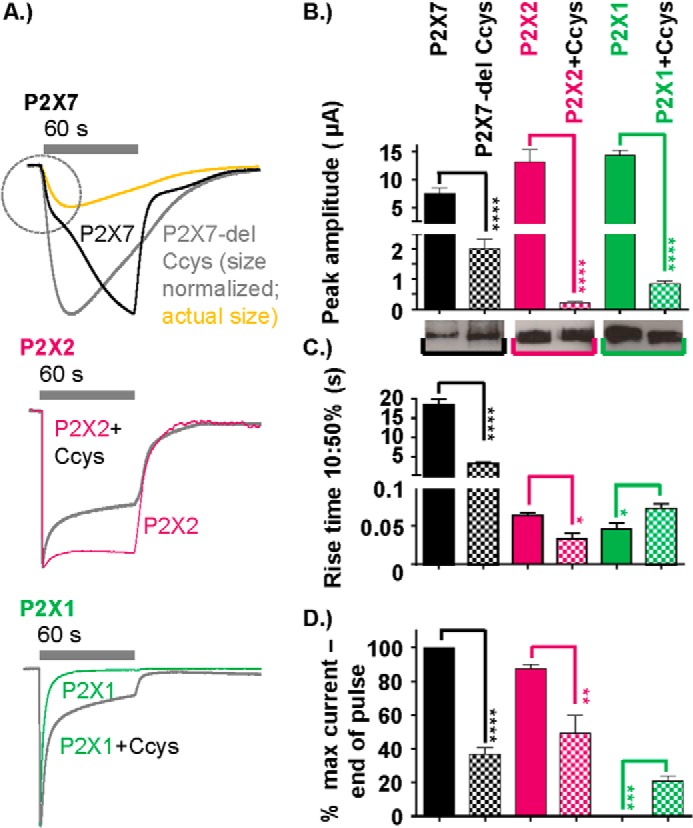
**Contribution of the hP2X7 receptor C-terminal cysteine-rich region (amino acid residues 362–379) to the time course.** *A*, peak normalized traces showing the effect on the time-course of cysteine-rich region deletion of the WT P2X7 receptor, insertion into the P2X2 receptor, and insertion into the P2X1 receptor. ATP application was as indicated by the *gray bars* (1 mm for P2X7 and P2X7–1Nβ and 100 μm for P2X1 and P2X2 WT receptors and chimeras). For the P2X7-delCcys, the trace in *yellow* shows the relative amplitude of the response compared with the P2X7 receptor to show the similar time course to the initial rise time of the P2X7 receptor response. *B—D*, histograms showing the peak current amplitude (microamperes), 10–50% rise time (seconds), and percent current remaining at the end of the ATP pulse. Western blots show equivalent surface expression of the respective WT and chimeric receptors (the WT control for P2X7 is the same as in Fig. 3, the original blot compared P2X7 WT with a range of P2X7 chimeras). Data are mean ± S.E. (*n* = 4–25). *, *p* < 0.05; **, *p* < 0.01; ***, *p* < 0.001; ****, *p* < 0.0001 (*n* = 4–25).

The P2X7 receptor is unique in having a cysteine-rich region (residues 362–379) just after TM2 and an extended C-terminal tail. Both removal of the cysteine-rich region and truncation of the C-terminal tail have been shown to have an effect on large pore formation (18, 28). However, the effects on current facilitation/time course are less well documented. Deletion of the cysteine-rich region after TM2 (P2X7-delCcys) resulted in an ∼6-fold speeding up of the time course of response to ATP and a reduction in peak current amplitude (∼25% of P2X7 WT), but this was not associated with a change in surface expression. Interestingly the rise time of the P2X7-delCcys mutant was equivalent to the initial phase of the P2X7 WT response (Fig. 4*A*). However, the current desensitized by ∼60% during the 60-s application. The peak current amplitude to a second application of ATP 5 min later was reduced to 3.9 ± 0.3% of the initial response (however, the time course was the same as the initial response). These results show that the Ccys region plays an important role in the generation of the slowly developing current at P2X7 receptors.

To test whether the Cycs region can independently result in a slowing of other P2X receptor currents, we inserted the cysteine-rich post-TM2 P2X7 region into P2X1 and P2X2 receptors (Fig. 4). Peak currents at these mutants were reduced by >90% (compared with the WT). This did not result from a change in receptor processing/trafficking because surface expression of the receptors was equivalent to WT P2X1 and P2X2 receptors (Fig. 4*B*). For P2X2 + Ccys, there was a modest, ∼2-fold speeding up of rise time and desensitization (Fig. 4). At the P2X1 receptor, the rate of desensitization was slowed (21 ± 3% and 0.2 ± 0.2% remaining at end of the 60-s pulse for P2X1 + Ccys and the WT, respectively). These results show that the P2X7 receptor C-terminal cysteine-rich region can be inserted into other P2X receptor channels and modifies channel gating but does not, on its own, impart a facilitatory phenotype.

##### Role of Interactions between Pre-TM1 and the P2X7 Receptor Ccys-rich Region

**FIGURE 5.**
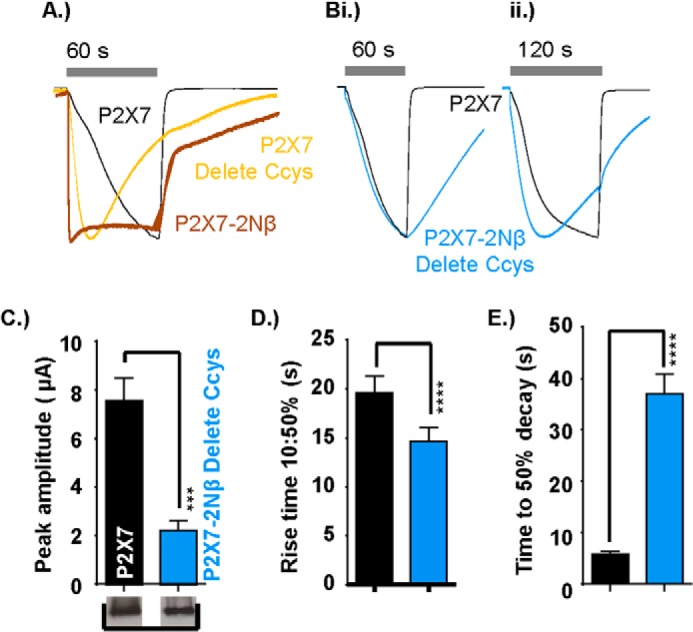
**Functional interaction between the pre-TM1 β-region and C-terminal cysteine-rich region and its effect on P2X7 receptor time course.** *A*, peak normalized traces showing the effect of deletion of the cysteine-rich region deletion from the P2X7 receptor (P2XYdelCcys, *yellow*) compared with the WT P2X7 receptor (*black*) and the P2X7–2Nβ chimera (*red*). ATP application is indicated by the *gray bars* (1 mm for 60 s). *B*, peak normalized traces showing reversion to slow receptor facilitation at the P2X7–2Nβ delCcys chimera (*blue*) with both 60-s (*i*) and 120-s (*ii*) applications of 1 mm ATP highlighting the faster desensitization of the chimeric receptor. *C–E*, histograms showing the peak amplitude (microamperes), rise time (seconds), and time to 50% decay (seconds) at the end of the ATP pulse for the P2X7 receptor *versus* the P2X7–2Nβ delCcys chimera. Western blots show equivalent levels of surface expression of P2X7 and the P2X7–2Nβ delCcys chimera. Data are mean ± S.E. (*n* = 4–25). ***, *p* < 0.001 ****, *p* < 0.0001.

This study has shown that, for the hP2X7 receptor, replacing the pre-TM1 Nβ region with that from the P2X2 receptor or removal of the Ccys region changed the time course of response and removed facilitation (Figs. 1, 4, and 5*A*). This raised the possibility that the intracellular amino and carboxyl termini regions interact to regulate the time course of hP2X7 receptors. To test this, we made a mutant (P2X7–2NβdelCcys) combining the replacement of the pre-TM1 region with deletion of the Ccys region (Fig. 5). The amplitude of ATP-evoked currents at the P2X7–2NβdelCcys was equivalent to those of the individual P2X7–2Nβdel and P2X7-delCcys mutants (and, compared with the hP2X7 receptor, reduced by ∼70%, Fig. 5*B*). Interestingly, when these two mutations were combined, the rise time of the ATP-evoked current was equivalent to the hP2X7 first response (18.4 ± 1.4 s and 14.6 ± 1.4 s for 10–50% rise time, respectively) and slower than either of the individual changes (P2X7–2Nβ or P2X7-delCcys, Fig. 5). However, in contrast to the P2X7 receptor, there was no speeding up of the second response to ATP (17.3 ± 1.9 s for 10–50% rise), the current desensitized during longer applications (percent of peak current remaining at the end of a 120-s pulse, 80.5 ± 0.7 and 100% for P2X7–2NβdelCcys and the WT respectively, *p* < 0.05), the current took longer to deactivate on ATP washout (Fig. 5*E*), and the peak amplitude was reduced by ∼50% (2.2 ± 0.4 μA and 1.0 ± 0.2 μA for the first and second response, respectively). These results show that interactions between the juxtatransmembrane regions regulate the time course of hP2X7 receptors and that time course and facilitation can be regulated independently.

##### Functional Measurements of P2X7 Receptor Pore Formation

**FIGURE 6.**
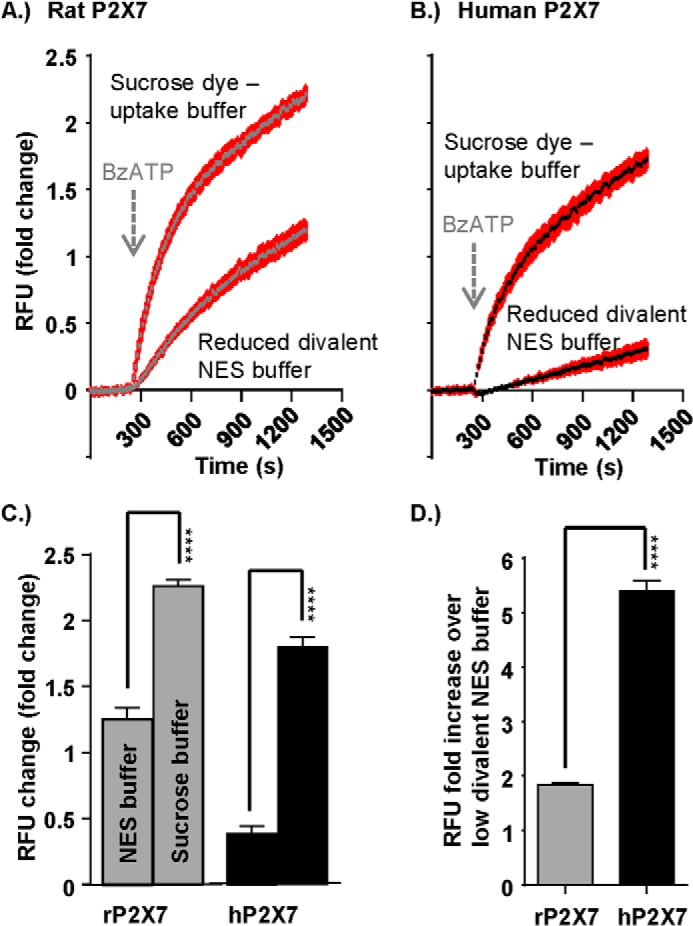
**Sucrose buffer enhances ethidium bromide dye uptake through the P2X7 receptor pore.** *A* and *B*, representative FlexStation responses showing agonist-induced dye uptake through the rat (*A*) and human (*B*) P2X7 receptor when preloaded for 30 min with ethidium bromide (20 μm) under different buffer conditions (sucrose dye uptake buffer *versus* normal extracellular solution low divalent buffer). Agonist addition (300 μm BzATP) at 240 s is indicated by the *arrow. C*, histogram summary showing the total RFU (relative fluorescence units) change under the different buffer loading conditions. *D*, histogram summary showing the -fold increase in dye uptake in sucrose dye uptake buffer compared with low divalent normal extracellular solution buffer. Data are mean ± S.E. ****, *p* < 0.0001 (*n* = 24).

P2X7 receptor pore dilation is not readily observed in oocytes (29, 30). Therefore, to characterize the effects on pore formation, P2X7 receptors were transiently expressed in HEK293-TSA201 cells, and agonist evoked ethidium bromide uptake was measured. In initial studies, we used a sodium chloride-based buffer (see “Experimental Procedures” and Fig. 6). The BzATP-evoked (300 μm) rises in ethidium bromide fluorescence for hP2X7 were 27% of those of rP2X7, similar to those reported previously (31, 32). Extracellular ions can have complex effects on P2X7 receptor gating (33, 34), native P2X7 receptor calcium responses were larger in a sodium-free solution (35), and a sodium-free sucrose buffer has been used in studies to assay hP2X7 receptors pore dilation (36). In sucrose (sodium-free) buffer, ethidium uptake (at 20 min) at rP2X7 receptors was increased ∼2-fold for rat P2X7 receptors compared with sodium buffer (*p* < 0.0001). A larger, ∼5.5-fold increase in uptake was seen for hP2X7 receptors when replacing sodium with sucrose (*p* < 0.0001 compared with rP2X7). In sucrose buffer, the level of EtBr uptake at 20 min was 78% of that for rP2X7 receptors. These results suggest that the smaller ethidium uptake of the hP2X7 in physiological solutions (compared with rP2X7) does not result predominantly from an inherent difference in the ability of the pore to dilate but is due to increased sensitivity to the inhibitory effects of sodium in the extracellular solution. Because of the increased ethidium uptake, we used sucrose buffer to characterize pore formation for the hP2X7 receptor mutants.

**FIGURE 7.**
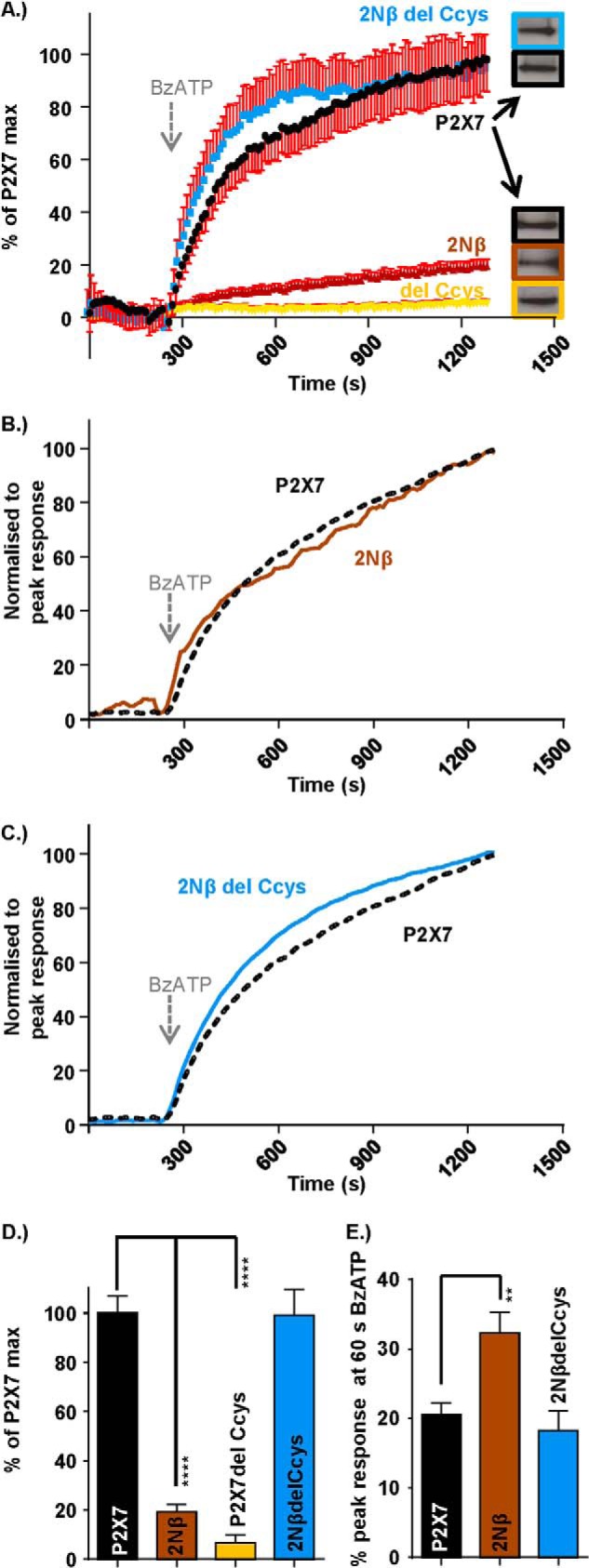
**Interaction of the intracellular pre-TM1 β region and C-terminal cysteine-rich region of the P2X7 receptor regulates pore formation and dye uptake.** *A*, FlexStation responses demonstrating agonist-induced dye uptake through the pore of the P2X7 receptor, reduced dye uptake though the N terminus chimeric receptor, and C terminus deletion receptor (P2X7–2Nβ and P2X7 delCcys, respectively) and “P2X7-like” dye uptake through the P2X7–2Nβ delCcys mutant receptor. Agonist addition (300 μm BzATP) at 240 s is indicated by the *arrow*. S.E. is only shown in one direction to not obscure the mean values. Western blots show equivalent levels of surface expression for the P2X7 receptor WT and mutants. *B*, P2X7–2Nβ receptor response normalized to the peak P2X7 receptor response identifying an increased initial rate of dye uptake. *C*, P2X7–2Nβ delCcys receptor response normalized to the peak P2X7 receptor response demonstrating the relative dye uptake speeds through these two receptors. *D*, histogram summary showing the relative dye uptake for each receptor. *E*, histogram summary demonstrating the amount of dye uptake (as a percentage of the peak maximum) 1 min after BzATP addition (300 μm BzATP addition made at 240 s). Data are mean ± S.E. **, *p* < 0.01; ****, *p* < 0.0001 (*n* = 7–21).

**FIGURE 8.**
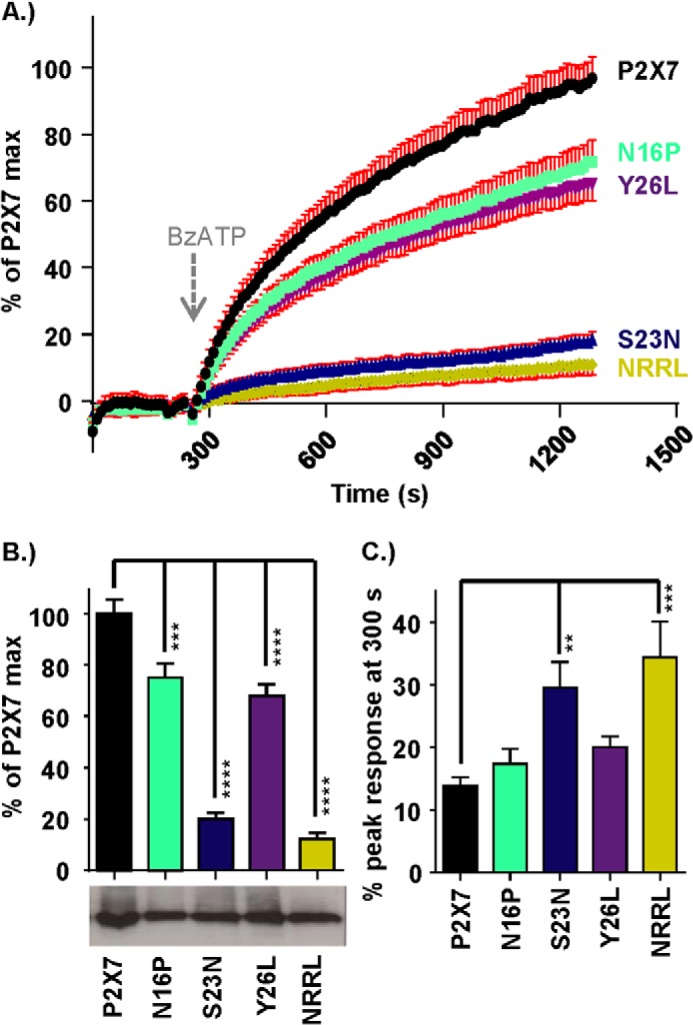
**Effects of mutations in the P2X7 receptor pre-TM1 β-region on pore formation and dye uptake.** *A*, FlexStation responses demonstrating agonist-induced dye uptake through the pore of the P2X7 receptor and reduced dye uptake though the N terminus mutants N16P, Y26L, S23N, and NRRL. Agonist addition (300 μm BzATP) at 240 s is indicated by the *arrow*. Standard errors are only shown in one direction to not obscure the mean values. *B*, histogram summary showing the relative peak dye uptake for each receptor. Western blots show equivalent levels of surface receptor expression for P2X7 receptor WT and mutants. *C*, histogram summary demonstrating the amount of dye uptake (as a percentage of the peak maximum) 1 min after BzATP addition (300 μm BzATP addition made at 240 s). Data are mean ± S.E. **, *p* < 0.01; ***, *p* < 0.001; ****, *p* < 0.0001 (*n* = 24).

At the P2X7–2Nβ chimera, ethidium uptake at 20 min was 12 ± 0.5% of that for hP2X7 receptors (surface expression levels of the chimera were equivalent to the hP2X7 WT) (Fig. 7). Ethidium uptake was also reduced significantly at mutants within the Nβ region that had an effect on the time course (N16P, NRRL, S23N, and Y26L; surface expression levels of the mutants were equivalent to the hP2X7 WT) (Fig. 8). For the mutants NRRL and S23N, which had a time course equivalent to P2X7–2Nβ (Fig. 2), the ∼90% reduction in dye uptake was similar to that seen for P2X7–2Nβ (Fig. 7). The initial rate of rise of the ethidium signal was faster for the P2X7–2Nβ than for the hP2X7 (percent response at 60 s as percent of peak 32.0 ± 3.1% and 20.5 ± 1.8%, respectively, *p* < 0.01). The rate of dye uptake was also significantly faster than hP2X7 for the NRRL and S23N mutants (Fig. 8). Taken together, these results show that P2X7 receptor mutants in which ATP-evoked currents were faster had a more rapid initial rate of ethidium uptake. However, this was not associated with a high total level of dye uptake.

Ethidium uptake at the hP2X7-delCcys receptor (an equivalent surface expression as hP2X7) was also significantly reduced by ∼95% compared with the hP2X7 receptor, consistent with previous studies (32) (Fig. 7). Surprisingly, when the Nβ domain swap and delCcys modifications were combined, the ethidium uptake returned to WT levels (Fig. 7). The results showing that independent modifications in the amino (Nβ) or carboxyl termini (Ccys) reduce ethidium uptake, but the combination of these modifications (P2X7–2NβCcys) then restores uptake, provides strong evidence that interaction between the juxtatransmembrane intracellular regions plays an important role in the regulation of large pore formation at P2X7 receptors.

## Discussion

This study shows, using a range of chimeras and point mutations, that both the amino and carboxyl terminus juxtatransmembrane regions are important for the regulation of hP2X7 receptor channel gating, contributing to both the novel facilitatory as well as the pore dilation phenotypes. Combining changes in the amino and carboxyl termini rescued the time course and large pore formation, suggesting that these regions interact to regulate the P2X7 receptor.

The slow rise time of hP2X7 receptor currents in this study is similar to that reported previously in oocytes (30) and HEK293 cells (20). The facilitation at the rP2X7 receptor has been modeled on the basis of changes in agonist binding (37, 38), is not due to changes/increases in surface expression (20), and has both calcium/calmodulin-dependent (C-terminal) and calcium-independent components (39). The carboxyl-terminal calmodulin binding motif is missing from hP2X7 receptors, and facilitation is not dependent on calcium (Ref. 20, this study was performed under calcium-free conditions). The return to the slow “naïve” hP2X7 receptor time course following a 10-min agonist-free period (this study) suggests that calcium-independent facilitation is readily reversible and results from a relatively short-lived conformational change.

The P2X7–2Nβ chimera shows that variant residues in the juxta-TM1 amino terminus region play an important role in determining the time course/facilitation of P2X7 receptor currents. The facilitation of hP2X7 receptor currents requires repeated long applications of agonist to evoke reproducible responses, and this leads to blebbing of the cells and pore dilation that can limit the time period over which responses can be recorded. At the P2X7–2Nβ chimeric receptor, reproducible responses to ATP were recorded readily and, because of the fast time course, steady-state currents could be recorded to brief 5-s agonist applications. We have also shown that the P2X7–2Nβ chimera has the same sensitivity as the hP2X7 receptor to a range of P2X7 receptor-selective antagonists.^3^ The P2X7–2Nβ chimera, therefore, has the potential to be useful for drug screening because it overcomes potential issues associated with the need to give repeated long applications of agonist to deal with the facilitatory nature of hP2X7 receptor currents.

Point mutations within the Nβ region identified residues associated with the speeding up of the current and removal of facilitation. It has been shown previously that the mutation of residues comprising the amino-terminal consensus sequence for protein kinase C can regulate the time course of currents and dilation of the P2X7 receptor pore (37). In other P2X receptor subunits, mutation of the consensus protein kinase C sequence also has marked effects on the channel time course, indicating the importance of this region in channel gating (40, 41). However, given that the consensus sequence is conserved in all P2X receptor subunits, other regions of the receptor must be involved in subtype-specific differences in time course/gating, *i.e.* for the slow time course and facilitation of the P2X7 receptor. In this study, we show that differences in the non-conserved residue within the (S/T)*X*(K/R) PKC consensus sequence (N16P) and at residue 23 (between the consensus PKC site and TM1) play an important role in P2X7 receptor channel gating. For N16P, the faster time course of the facilitated current was not associated with a reduction in peak amplitude, and an asparagine residue at this position is unique to the P2X7 receptor. Interestingly, at the P2X2 receptor, currents at a cysteine substitution mutant of the corresponding proline residue were reduced by 70% by treatment with the methyl methanethiosulfonate (MTSM) (42). In addition, cysteine mutants of residues equivalent to R20,I21 (V23,V24 in P2X2) were also inhibited by 70–100% by MTSM (42), suggesting that this region contributes to ionic permeation. Further evidence for a regulatory role of this region comes from recent work on hP2X7 receptors showing that Lys-17 and Val-18 are involved in cholesterol sensitivity (32). Taken together, a range of studies have highlighted the important role of variant residues in the pre-TM1 region in regulation of ion channel activity. However, the P2X7 receptor pre-TM1 region did not confer the facilitatory phenotype when the region was swapped in reciprocal chimeras with P2X1 and P2X2 receptors (where responses were faster and showed increased desensitization compared with the WTs). This suggests that the amino-terminal juxta-TM1 region does not act independently but regulates gating via interaction with other parts of the receptor.

The deletion of the Ccys region from the P2X7 receptor had no effect on the initial rate of rise of the ATP-evoked current (normalized traces were superimposable, Fig. 4). However, the current then desensitized and did not have the slowly developing secondary component. This desensitizing phenotype corresponds to that described for activation of naïve receptor by low agonist concentrations that has been modeled to occupancy of one or two ATP-binding sites at the naïve receptor (38). Occupancy of the third ATP binding site then favors the transition to the secondary developing current (38). Our data show that deletion of the Ccys region impairs the normal gating transitions required for the secondary component. The Ccys region could be introduced into both P2X1 and 2 receptors with only modest effects on their time courses (slowing and speeding up, respectively). The Ccys region has been shown to be involved in cholesterol sensitivity at P2X7 receptors, and it has been proposed that the proximal C-terminal region is “in contact with or may dip back into the plasma membrane” (32). The inclusion of the Cys region in P2X1 and 2 receptors could, therefore, provide an “additional membrane anchor” of the C-terminal region that accounts for the effects on time course. The ability to swap the Ccys region into P2X1 and 2 receptors with only modest effects on the time course suggests that it does not independently confer the slow secondary growth in current.

The P2X7–2Nβ chimera (and the S23N and NRRL mutants) as well as P2X7-delCcys mutants had effects not only on the time course of agonist-evoked currents but also reduced ethidium uptake by ∼90–95%, indicating a significant effect of disrupting both of these regions on pore dilation. The effects of the Ccys deletion are consistent with a recent study of the hP2X7 receptor showing reduced large pore formation when the region was deleted (32). However, at the rP2X7 receptor, the rate of dye uptake was enhanced following Ccys deletion (28), indicating species differences in their regulation/properties. To our surprise, combination of mutations in the N and C juxtamembrane regions (P2X7–2NβdelCcys) rescued ethidium uptake and the rise time of peak current to WT levels. The mutation of both regions does not return the receptor completely to WT properties because the current showed some desensitization and was slower to deactivate following agonist removal, and peak current amplitudes were reduced by ∼75%. These results (and those from other chimeras) highlight that there is no clear correlation between the peak current amplitude, kinetics, and pore formation, suggesting that the processes are not interdependent. These results also indicate that the P2X7-specific Nβ and Ccys regions (absent in P2X7–2NβdelCcys) are not required for pore dilation. This is consistent with the pore dilation observed at P2X2&4 receptors that vary in their amino-terminal region and lack the Ccys insertion (43, 44) and suggests that other parts of the receptor underlie pore dilation.

The rescue of dye uptake and rise time at P2X7–2NβdelCcys suggests that there is a complex interaction between the N and C-terminal regions. Replacement of the Nβ region of P2X7 with P2X2 limited pore dilation, indicating that there is some other region of the receptor that imposes an inhibitory effect. Similarly, removal of the Ccys region also limits pore dilation, indicating another inhibitory region of the receptor. One possibility is that there was a coevolution of the amino and carboxyl-terminal regions to complement one another. That is, either region could exert an inhibitory effect on pore dilation on their own, but, when combined, they cancel the effects of each other and allow for pore dilation. For example, replacement of the Nβ region of P2X7 with P2X2 removed the positive regulatory interaction between the amino and carboxyl-terminal regions with the net result of the inhibitory effect of the Ccys region dominating the dilation phenotype. Similarly, deletion of the Ccys region removed the regulatory interaction with the amino-terminal Nβ region and resulted in the net inhibitory effect of the P2X7 receptor Nβ region. Because the intracellular regions of the zebrafish P2X4 receptor were truncated to aid crystallization, there are no structural data on the organization of the intracellular regions of P2X receptors (45, 46) to provide a template for modeling potential interactions in the P2X7 receptor.

In summary, we show that both the juxtamembrane intracellular amino and carboxyl termini of the hP2X7 receptor are involved in the regulation of time course and pore dilation. Our results suggest a complex interaction between the intracellular termini in the regulation of channel properties. These regions are some distance from the pore gate and demonstrate that the intracellular regions exert considerable regulatory control on the movement of the transmembrane helices associated with channel opening.

## Footnotes

3 R. C. Allsop and R. J. Evans, unpublished observations.

## Footnotes

2 The abbreviations used are:

TM: transmembrane
Ccys: cysteine-rich
BzATP: 2′,3′-*O*-(4-benzoylbenzoyl)-ATP
MTSM: methyl methanethiosulfonate.
